# Identification of Key Genes With Differential Correlations in Lung Adenocarcinoma

**DOI:** 10.3389/fcell.2021.675438

**Published:** 2021-05-05

**Authors:** You Zhou, Bin Xu, Yi Zhou, Jian Liu, Xiao Zheng, Yingting Liu, Haifeng Deng, Ming Liu, Xiubao Ren, Jianchuan Xia, Xiangyin Kong, Tao Huang, Jingting Jiang

**Affiliations:** ^1^Tumor Biological Diagnosis and Treatment Center, The Third Affiliated Hospital of Soochow University, Changzhou, China; ^2^Jiangsu Engineering Research Center for Tumor Immunotherapy, Changzhou, China; ^3^Institute of Cell Therapy, Soochow University, Changzhou, China; ^4^Department of Immunology and Biotherapy, Tianjin Medical University Cancer Institute and Hospital, Tianjin, China; ^5^State Key Laboratory of Oncology in South China, Collaborative Innovation Center for Cancer Medicine, Sun Yat-sen University Cancer Center, Guangzhou, China; ^6^CAS Key Laboratory of Tissue Microenvironment and Tumor, Shanghai Institute of Nutrition and Health, Chinese Academy of Sciences, Shanghai, China; ^7^Bio-Med Big Data Center, CAS Key Laboratory of Computational Biology, Shanghai Institute of Nutrition and Health, Chinese Academy of Sciences, Shanghai, China

**Keywords:** WGCNA, differential correlation, switching mechanism, gene regulation, lung adenocarcinoma

## Abstract

**Background:**

With the advent of large-scale molecular profiling, an increasing number of oncogenic drivers contributing to precise medicine and reshaping classification of lung adenocarcinoma (LUAD) have been identified. However, only a minority of patients archived improved outcome under current standard therapies because of the dynamic mutational spectrum, which required expanding susceptible gene libraries. Accumulating evidence has witnessed that understanding gene regulatory networks as well as their changing processes was helpful in identifying core genes which acted as master regulators during carcinogenesis. The present study aimed at identifying key genes with differential correlations between normal and tumor status.

**Methods:**

Weighted gene co-expression network analysis (WGCNA) was employed to build a gene interaction network using the expression profile of LUAD from The Cancer Genome Atlas (TCGA). R package DiffCorr was implemented for the identification of differential correlations between tumor and adjacent normal tissues. STRING and Cytoscape were used for the construction and visualization of biological networks.

**Results:**

A total of 176 modules were detected in the network, among which yellow and medium orchid modules showed the most significant associations with LUAD. Then genes in these two modules were further chosen to evaluate their differential correlations. Finally, dozens of novel genes with opposite correlations including ATP13A4-AS1, HIGD1B, DAP3, and ISG20L2 were identified. Further biological and survival analyses highlighted their potential values in the diagnosis and treatment of LUAD. Moreover, real-time qPCR confirmed the expression patterns of ATP13A4-AS1, HIGD1B, DAP3, and ISG20L2 in LUAD tissues and cell lines.

**Conclusion:**

Our study provided new insights into the gene regulatory mechanisms during transition from normal to tumor, pioneering a network-based algorithm in the application of tumor etiology.

## Introduction

Lung cancer has been one of the most leading causes responsible for cancer mortality globally for several decades (Halliday et al., 2019). According to the Surveillance, Epidemiology, and End Results (SEER) Program, 228,820 new cases and 135,720 deaths of lung and bronchus cancer patients have been estimated in the United States in 2020 (Siegel et al., 2020). In China, estimated new cases and deaths from lung cancer were 730,000 and 610,000 in 2015, respectively, accounting for over 30% of the world’s total (Chen et al., 2016). Based on histological subtypes, approximately 85% are of non-small cell lung cancer (NSCLC), of which lung adenocarcinoma (LUAD) is the most prevalent and attributed to more than 50% of all lung cancer cases (Oberndorfer and Mullauer, 2018). Despite that conventional therapies including surgery, radiotherapy, chemotherapy, immunotherapy, and targeted drugs have been applied to LUAD treatment, the 5-year survival rate for patients remains less than 15%, mainly due to lack of early detection and intervention (Herbst et al., 2018). In addition to environmental exposures and tobacco smoking, genetic susceptibility has been recognized as the most important risk factor associated with LUAD. Recently, a new conception called “oncogene addiction” referring to dependence of cancer cells on the activation of specific oncogenes has attracted increasing attention and underlined the significance of oncogenic drivers in LUAD (Calvayrac et al., 2017). At present, patients with LUAD harboring gene aberrations in EGFR (Thomas et al., 2019), KRAS (Kim et al., 2019), ALK (Shaw et al., 2019), BRAF (Dankner et al., 2018), HER2 (Mazieres et al., 2013), and MET (Drilon et al., 2017) benefit the most from targeted therapies, and those genes have been considered as oncogenic drivers in the field of thoracic oncology. However, on account of the dynamic mutational spectrum comprised of a vast number of hidden drivers, only a minority of patients archived improved outcome under current standard therapies (Oberndorfer and Mullauer, 2018). Thus, it is imperative to identify new risk genes for elucidating lung carcinogenetic mechanisms in order to guide researchers to develop new therapeutic strategies and physicians to tailor the treatment options.

Exponential advances in the high-throughput sequencing technologies and informatics have generated large-scale omics data which promotes a paradigm shift in the study of biomedical sciences and is available for deciphering molecular characteristics of oncogenesis that could be translated into clinical practice (Manzoni et al., 2018; Cheng, 2020). Among them, transcriptome data is quite informative because of the discovery of the significantly altered abundance of cellular components or pathways between disease and healthy tissues or two disease states (Kwon et al., 2019). In recent years, comparative analysis of transcriptome data has helped to discover prognostic markers and signatures by looking for differentially expressed genes in a variety of cancer types (Jiang et al., 2015; Zhang et al., 2020). However, a fact that could not be ignored is that most of the biological activities require an orchestrated action of multiple genes, whose dysregulation could lead to the occurrence of cancer. Therefore, gene correlation approaches have been intensively used for transcriptional profiling, providing preliminary steps toward genetic interaction networks and offering clues about the function of unknown genes.

Complementary to gene correlation analysis, changes in correlation patterns under different conditions, referred to as “differential correlations,” are also attractive as for the reconstruction of genome architecture and identification of regulators or marker genes (Ideker and Krogan, 2012; Li et al., 2015). For example, one transcription factor defined as a master gene regulates a number of downstream targets. Also, these targets express in an ordered module where the regulatory mechanism is functional. However, in disease tissues where the regulatory mechanism is malfunctional, the gene expression module may be disordered or random. In this case, correlation changes can be detected by differential correlations rather than differential expression analysis. Recently, such alterations in network structures and variance in gene expression levels have been observed in cellular differentiation and cancer cell formation (Bockmayr et al., 2013; Ando et al., 2015), contributing to understanding the expression patterns between two states. However, differential correlations in LUAD are poorly determined, so it is imperative to unravel network dynamics that can be used to identify new candidate genes. Hitherto, an increasing number of systematic approaches have been developed for identifying such changes in network structures (Ideker and Krogan, 2012; Yu et al., 2015). Application of these methods to LUAD and adjacent normal tissues may reveal variance in gene expression levels during carcinogenesis.

**FIGURE 1:**
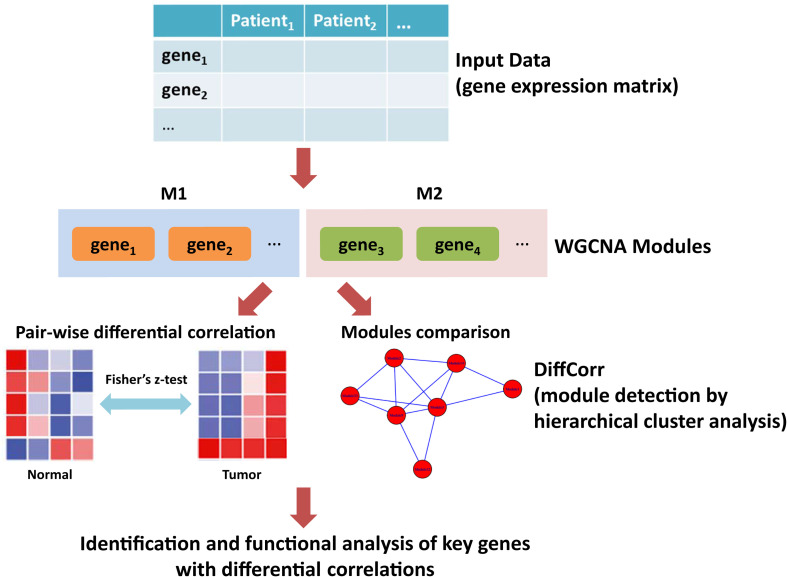
The workflow of this study. First, the gene expression matrix of LUAD patients was obtained. Second, the WGCNA network was constructed and genes were clustered into modules. Third, differential correlations between genes were calculated and significant differences between tumor and adjacent normal tissues were identified using DiffCorr. Finally, functional analysis of key genes with differential correlations.

Weighted gene co-expression network analysis (WGCNA) is a systematic method widely utilized in oncology research that aims at finding co-expressed genes through calculating gene connectivity (Chang et al., 2013; Yang et al., 2018). The soft thresholding of the Pearson correlation matrix is integrated into analysis for determining the connection strengths within gene pairs (Zhang and Horvath, 2005; Ghazalpour et al., 2006). Based on Fisher’s *z*-test, R package DiffCorr is a useful tool in identifying differential correlations between disease states and providing a list of differentially correlated gene pairs (Fukushima, 2013). In this study, we developed an *in silico* framework to identify key genes with differential correlations (Figure 1). First, we employed WGCNA to build a gene interaction network using the expression profile of LUAD from The Cancer Genome Atlas (TCGA). A total of 176 modules were detected in the network, among which yellow and medium orchid modules showed the most significant associations with LUAD. Then, we calculated differential correlations of genes in these two modules and identified significant differences between tumor and adjacent normal tissues using DiffCorr. Finally, dozens of new genes including ATP13A4-AS1, HIGD1B, DAP3, and ISG20L2, were identified and their expression patterns were confirmed by real-time qPCR. Further biological and survival analysis yielded their valuable effects on the progression of LUAD.

## Materials and Methods

### LUAD RNA-Sequencing Datasets

The RNA-sequencing data of LUAD was downloaded from the TCGA database^1^, including 468 tumor samples and 58 normal samples. As previously described, the gene expression levels were quantified as FPKM (fragments per kilobase per million mapped reads) using TopHat and HTSeq-count (Kim et al., 2013; Anders et al., 2015). The TCGA sample information was listed in Supplementary Table 1. Within the 468 tumor samples, 3 samples did not have stage information, 165 samples were in stage I, 247 samples were in stage II, 39 samples were in stage III, and 14 samples were in stage IV. The sample distribution also stressed the importance of identification of new genes for early diagnosis.

### Co-expression Network Analysis

Hub gene screening and co-expression of gene pair detection were performed by an R package WGCNA, which was designed for analyzing a weighted correlation network (Langfelder and Horvath, 2008). The premise of the network construction was that elements in the gene co-expression matrix were the weighted values of the correlation coefficient between gene pairs. The selection criterion of the weight was to make the connections among genes conform to the scale-free network distribution; that is, for the number of connections *i*, the probability *p(i)* was inverse to *i*^*n*^. Practically, a weighted coefficient was selected to approximate the scale-free topology, which needs to satisfy the following condition: the log of the number of connected nodes *log(i)* was negatively correlated with the log of occurrence probability of nodes *log(p(i))* (the correlation coefficient should be at least 0.8). At the same time, the average connection degree of genes in different modules should be quite high. Associated genes were clustered based on dissimilarity of the unsigned topological overlap matrix (TOM). Finally, network modules and the genes within them were identified.

### Functional Enrichment Analysis of Module Genes

R package clusterProfiler was used to perform functional enrichment analysis on clustered genes in yellow and medium orchid modules. A hypergeometric distribution test was applied to detect enrichment terms, and *P* values were adjusted by false discovery rate (FDR) method with a cutoff FDR < 0.05 (Yu et al., 2012).

### Differential Correlation Analysis

R package DiffCorr was implemented for the identification and visualization of differential correlations in biological networks, and details were described in Fukushima (2013). Briefly, the DiffCorr package mainly contained three functions. First is calculation of differential correlations. The correlation coefficients for each of the two conditions (herein referred to tumor and normal), *r*_*A*_ and *r*_*B*_, were transformed into *Z*_*A*_ and *Z*_*B*_, respectively, based on Fisher’s transformation: $Z=\frac{1}{2}\text{log}\frac{1+\text{r}}{1-r}$. Differences between the two correlations could be tested using the equation $Z=\frac{Z_{A}-Z_{B}}{\sqrt{\frac{1}{n_{A}-3}+\frac{1}{n_{B}-3}}}$, where *n*_*A*_ and *n*_*B*_ represent the sample size for each biomolecule pair in each condition. Then, the local false discovery rate (lfdr) derived from the fdrtool package was used for controlling true estimates and identifying significant differential correlations. Second is identifying eigen-molecules. Eigen-molecules or “eigengenes” in the network were calculated based on the first principal component of a data matrix of a module which was extracted from a hierarchical cluster analysis. In addition to pair-wise differential correlations between molecules, differential correlations between modules were tested by using these eigen-molecule modules. Third is scaling and clustering. Different pretreatment methods with downstream correlation analyses were integrated, including auto-scaling, range scaling, Pareto scaling, vast scaling, level scaling, and power transformation.

### Visualization and Functional Analysis

Construction and analysis of networks were carried out using STRING (11.0)^2^ (Szklarczyk et al., 2019). Visualization of networks was realized using Cytoscape (3.6.0) (Doncheva et al., 2019).

### The Survival Analysis of Key Genes With Differential Correlations

There were 468 LUAD patients with survival information. We performed survival analysis using the Cox proportional hazard regression model on these samples (Andersen and Gill, 1982). Each gene expression level was divided into two groups and repeated for 90 times based on the cutoff value from 5 to 95% of its expression level. A repeated log-rank test based on each cutoff value was processed, and a cutoff value with the lowest *P*-value was selected for subsequent univariate survival analysis. The Kaplan–Meier plot was used to describe the survival curves of these two groups of patients. The significance of the survival difference between these two patient groups was evaluated by the log-rank test *P* value. If the *P* value was less than 0.05, its survival was considered as significantly different. The R package survival was used to perform the survival analysis.

### Cell Lines and Cell Culture

The LUAD cell lines (A549, SPCA1, PC9, H1299, and H1975) and the normal human bronchial epithelial cell line 16HBE were from the Cell Bank of Type Culture Collection of the Chinese Academy of Sciences, Shanghai Institute of Cell Biology (Shanghai, China). A549, H1975, and H1299 cells were grown in RPMI-1640 (Thermo, United States) containing 10% FBS, and 16HBE, SPCA1, and PC9 cells were grown in a DMEM medium (Thermo, United States) containing 10% fetal bovine serum (FBS). In addition, all cell media were supplemented with penicillin (100 U/mL) and streptomycin (100 U/mL) at 37°C in a 5% CO_2_ incubator.

### LUAD Patients and Tissue Specimens

A total of 15 pairs of LUAD tumor and corresponding adjacent normal tissues were collected from the Third Affiliated Hospital of Soochow University between October 2019 and June 2020. Samples were snap-frozen and stored at −80°C until use in real-time qPCR (RT-qPCR) experiments. In addition, we conducted immunohistochemical staining of formalin-fixed paraffin-embedded LUAD patients and normal control samples. The Research Ethics Committee of the Third Affiliated Hospital of Soochow University approved this study, which was consistent with the Declaration of Helsinki. All patients provided written informed consent.

### RNA Isolation, cDNA Synthesis, and RT-qPCR

We isolated total RNA with TRIzol reagent (Thermo, United States). First-strand cDNA was synthesized from 1 μg total RNA using a ReverTra Ace qPCR RT Kit (Toyobo, Osaka, Japan). The Fast SYBR Green Master Mix was used for RT-qPCR (Applied Biosystems Inc., CA, United States). The cycling conditions were 30 s of polymerase activation at 95°C followed by 40 cycles at 95°C for 5 s and 60°C for 30 s. GAPDH was used as an internal loading control. The relative level was calculated by the relative quantification 2-ΔΔCT method. All primer sequences were listed in Supplementary Table 2.

### Statistical Analysis

Student’s *t*-test was applied to identify genes differentially expressed between normal and tumor samples. *P* values were adjusted by the Benjamini–Hochberg method (Hochberg and Benjamini, 1990). Differentially expressed genes were defined as adjusted *P* value < 0.05. Fisher’s *z*-test was employed to evaluate differential correlations of gene pairs between normal and tumor samples. Moreover, lfdr < 0.05 was defined as significant differential correlations.

## Results

### Construction of the Co-expression Network

**FIGURE 2:**
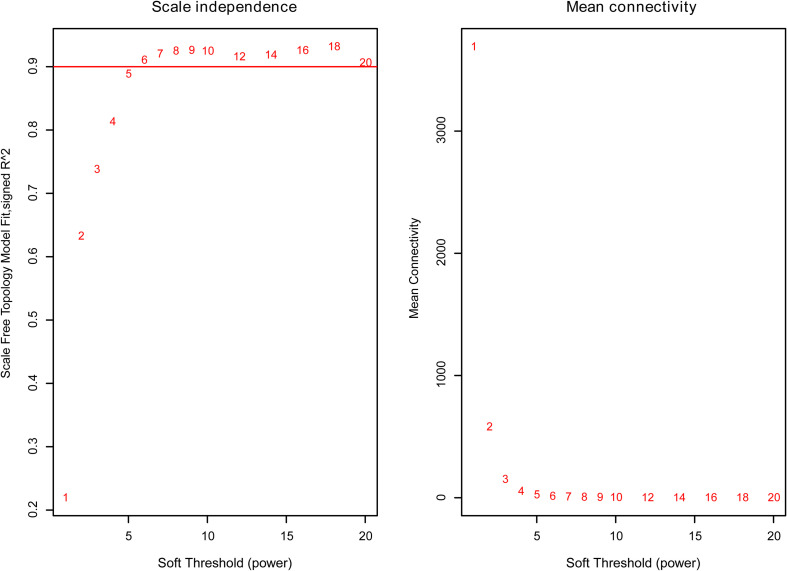
The relationship between soft threshold (power) and network properties. Left panel: The relationship between soft-threshold (power) and scale-free topology. Right panel: The relationship between soft threshold (power) and mean connectivity. When the soft threshold (power) was six, the scale-free topology (*R*^2^) was 0.91 and mean connectivity became stable. Therefore, we set the soft threshold (power) to be six.

**FIGURE 3:**
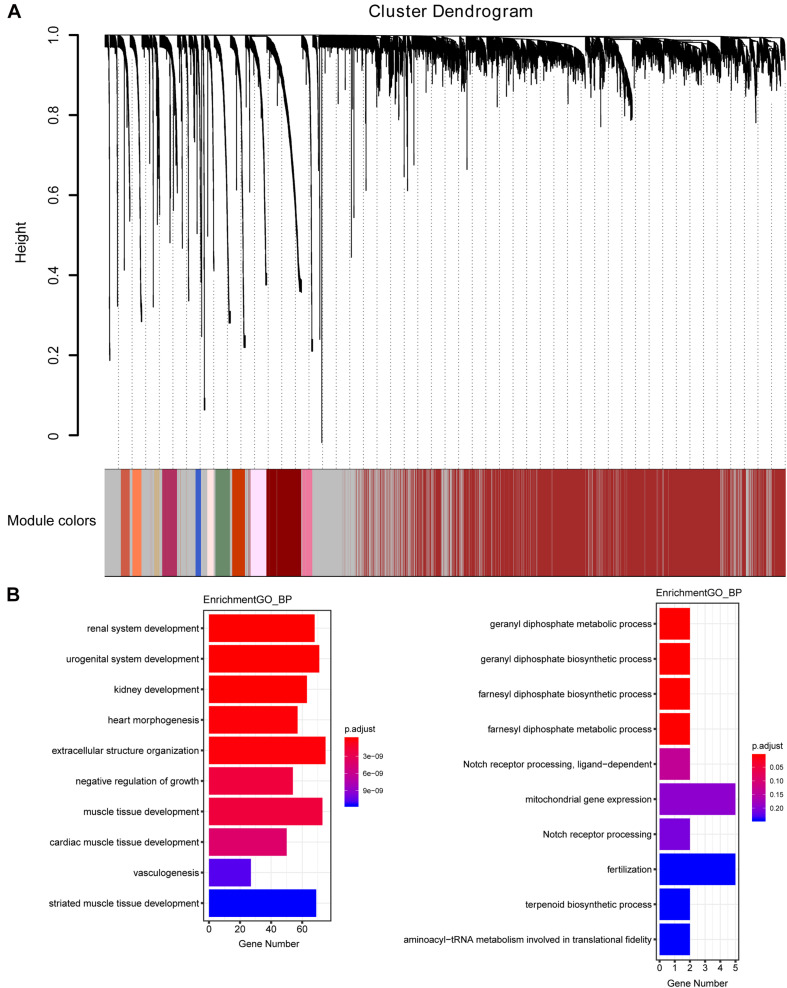
The cluster dendrogram of the WGCNA co-expression network and functional enrichment of module genes. **(A)** Total genes were clustered in 176 modules. Each module was marked with one color. Except for the gray module, which included many unclassified members, the turquoise module contained a maximum of 4,594 members, while a minimum of 30 members were included in the dark sea green module. **(B)** GO analysis showed the top 10 enriched biological processes in the yellow (left panel) and medium orchid (right panel) modules.

The weighted gene co-expression network was constructed from 58,387 coding and noncoding RNAs through the WGCNA approach. Here, the soft-thresholding power was set to be six to satisfy the scale-free topology of the network (Figure 2), in which R^2^ was used to check how well the network fit the scale freeness. When the soft-thresholding power was set to be six, the R^2^ was 0.91. Furthermore, we detected 176 modules in this network, whose relationship was shown in a cluster dendrogram (Figure 3A). The number of members in different modules varied widely. The members of each module were listed in Supplementary Table 3. Besides the gray module which comprised many unclassified members, the turquoise module contained a maximum of 4,594 genes, while a minimum of 30 genes were included in the dark sea green module.

Each module represented a group of genes with similar expression profiles across samples. Next, we quantified module-trait associations (Supplementary Figure 1), among which the yellow and medium orchid modules showed the most significant associations with LUAD. The corresponding correlation coefficients of yellow and medium orchid modules were 0.83 (*P* = 410^–133^) and −0.58 (*P* = 510^–49^), respectively. Clearly, Gene Significance (GS) and Module Membership (MM) analysis illustrated that genes highly significantly associated with LUAD were also the most important elements of modules associated with LUAD (Supplementary Figure 2). We also performed Gene Ontology (GO) enrichment analysis of these two modules. As presented in Figure 3B, genes in the yellow module were significantly enriched in the negative regulation of growth and vasculogenesis, while genes in the medium orchid module were enriched in the cellular metabolic process (geranyl diphosphate metabolic process, geranyl diphosphate biosynthetic process, farnesyl diphosphate biosynthetic process, and farnesyl diphosphate metabolic process) with an adjusted *P* value smaller than 0.05, conferring the importance of these biological functions on LUAD development.

### Identification of Differential Correlations

**FIGURE 4:**
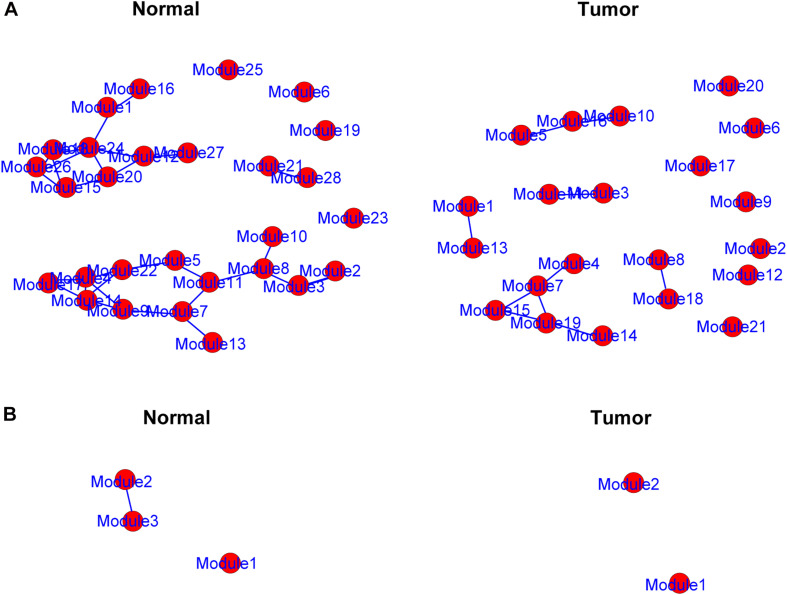
Representation of the module networks. Images of yellow **(A)** and medium orchid **(B)** module networks from the TCGA dataset were shown. Each node represented one module, and each edge represented the module correlation.

The genes in the yellow and medium orchid modules were further chosen to evaluate their differential correlations. Using R package DiffCorr, these genes were grouped based on their expression patterns in each subtype (normal or tumor) using the *cluster.molecule* function. We used the one-correlation coefficient as a distance measure (the cutoff of the coefficient was 0.6) according to the *cutree* function. The *get.eigen.molecule* and *get.eigen.molecule.graph* functions were used for visualization of the module network (Figure 4). The *comp.2.cc.fdr* function provided the resulting pair-wise differential correlations in the yellow (Supplementary Table 4) and medium orchid modules (Supplementary Table 5).

**FIGURE 5:**
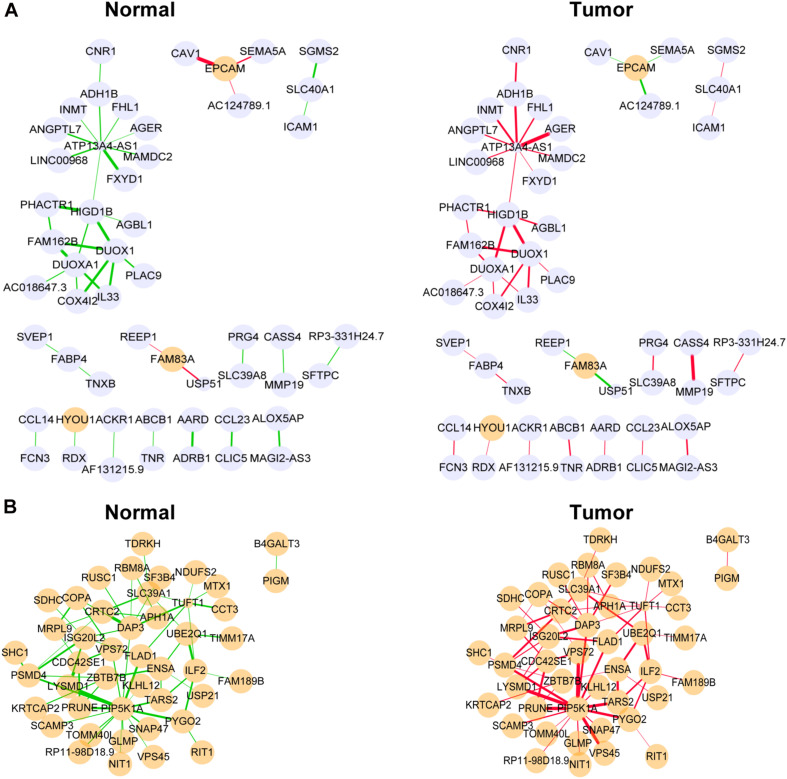
Differentially co-expressed gene networks in the yellow **(A)** and medium orchid **(B)** modules from the TCGA dataset. Each node represented a gene, with lavender-filled color denoting downregulation and orange-filled color upregulation. The larger size of node represented smaller adjusted *P*-value. The edge represented connection between two genes. The green edge represented negative correlation and red positive correlation. The thicker part of the edge represented a stronger correlation coefficient.

The DiffCorr package also detected oppositely correlated pairs where, for example, two genes exhibited a positive correlation in normal samples and a negative correlation in tumor samples, or *vice versa*, a condition referred to as a “switching mechanism” (Kayano et al., 2011). These switched gene pairs, which especially show a differential expression at the same time, were worth noticing for their critical roles in understanding cellular functions in the development of LUAD. In total, we obtained 42 oppositely correlated gene pairs with a differential expression simultaneously from the yellow module and 62 from the medium orchid module (Supplementary Table 6). Their interaction networks are shown in Figure 5. The top 10 significant switching mechanisms of gene expression between normal and tumor samples from the yellow and medium orchid modules are shown in Table 1.

**TABLE 1:** Top 10 correlated gene pairs changed to the opposite direction from the yellow and medium orchid modules between normal and LUAD samples.

Molecule X	Molecule Y	r1 (normal)	r2 (tumor)	lfdr^*a*^ (difference)	Module color
HIGD1B	DUOX1	–0.58	0.53	0	Yellow
FAM162B	DUOX1	–0.56	0.51	0	Yellow
EPCAM	CAV1	0.65	–0.40	0	Yellow
FAM162B	DUOXA1	–0.59	0.46	4.67E-12	Yellow
PHACTR1	HIGD1B	–0.58	0.45	1.47E-11	Yellow
DUOX1	COX4I2	–0.55	0.47	3.16E-11	Yellow
IL33	DUOX1	–0.53	0.48	8.28E-11	Yellow
FXYD1	ATP13A4-AS1	–0.58	0.41	1.29E-10	Yellow
MMP19	CASS4	–0.43	0.55	2.30E-10	Yellow
ATP13A4-AS1	AGER	–0.40	0.58	2.51E-10	Yellow
VPS72	PIP5K1A	–0.47	0.73	0	Mediumorchid
VPS45	PIP5K1A	–0.41	0.69	0	Mediumorchid
UBE2Q1	ILF2	–0.60	0.58	0	Mediumorchid
TARS2	PIP5K1A	–0.54	0.66	0	Mediumorchid
TARS2	ENSA	–0.46	0.62	0	Mediumorchid
PYGO2	PIP5K1A	–0.59	0.62	0	Mediumorchid
PSMD4	PIP5K1A	–0.55	0.60	0	Mediumorchid
PRUNE	PIP5K1A	–0.58	0.70	0	Mediumorchid
PRUNE	CDC42SE1	–0.55	0.59	0	Mediumorchid
PIP5K1A	LYSMD1	–0.74	0.71	0	Mediumorchid

*^a^Local FDR.*

### Functional Analysis of Key Genes With Differential Correlations

**FIGURE 6:**
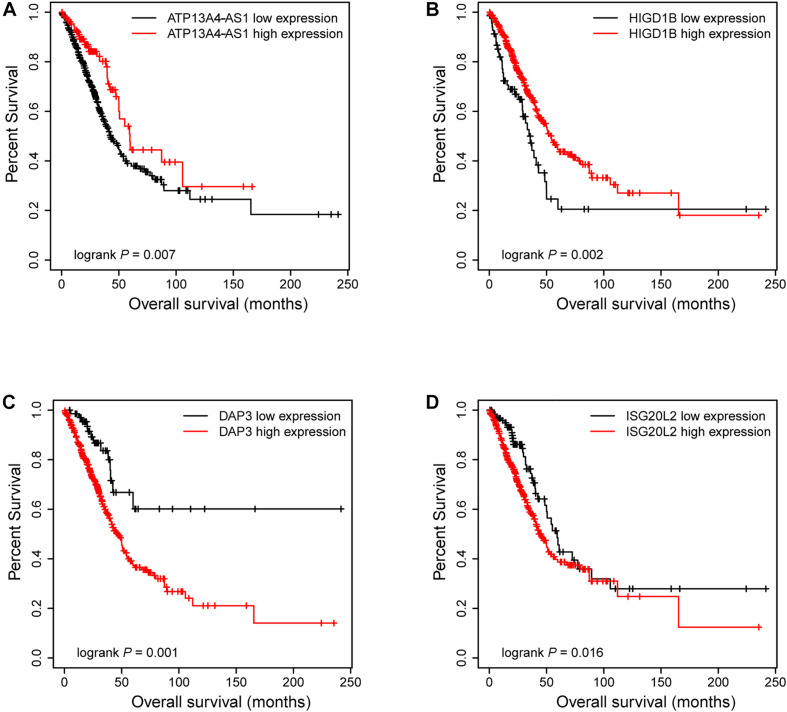
The Kaplan–Meier plot of ATP13A4-AS1, HIGD1B, DAP3, and ISG20L2. The low expressions of ATP13A4-AS1 **(A)** and HIGD1B **(B)** were associated with high risk. The high expressions of DAP3 **(C)** and ISG20L2 **(D)** were associated with high risk.

**FIGURE 7:**
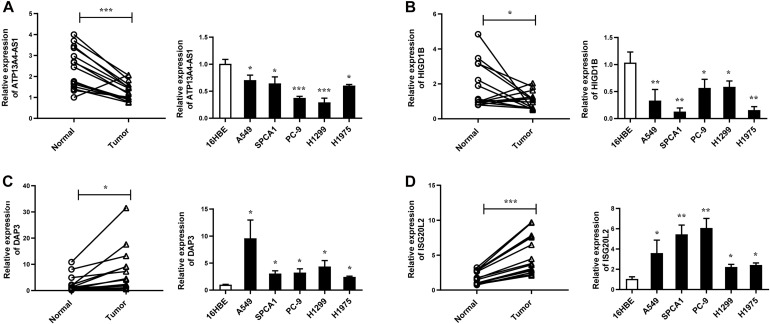
The expression levels of ATP13A4-AS1 **(A)**, HIGD1B **(B)**, DAP3 **(C)**, and ISG20L2 **(D)** in LUAD tissues and cell lines detected by RT-qPCR. Human bronchial epithelial cell line: 16BE. LUAD cell lines: A549, SPCA1, PC9, H1299, and H1975. Data were means ± SEM. ^∗^*P* < 0.05, ^∗∗^*P* < 0.01, ^∗∗∗^*P* < 0.001. Experiments were repeated three times.

Next, we focused on several key genes that acted as master regulators for their occupying most connections with other genes. In the yellow module, DUOX1, DUOXA1, HIGD1B, and ATP13A4-AS1 stood out in the network (Figure 5A). ATP13A4 antisense RNA 1 (ATP13A4-AS1) is an antisense long noncoding RNA (lncRNA) derived from ATP13A4 with an unknown function in cancer. Here, we can infer the roles of ATP13A4-AS1 from its interacted genes advanced glycosylation end-product specific receptor (AGER) and angiopoietin-like 7 (ANGPTL7) (Figure 5A). As a member of the immunoglobulin superfamily, cell surface receptor AGER is involved in multiple inflammatory responses whose abnormal expression has been reported to be closely associated with carcinogenesis (Bongarzone et al., 2017). Accumulating evidence has shown the downregulation of AGER in lung cancer, leading to enhanced proliferation, invasion and migration abilities, and decreased apoptosis of cells (Zhang et al., 2018; Wang et al., 2020). In addition, genetic polymorphisms of AGER have been reported to increase risks of lung cancer and breast cancer (Yin et al., 2015; Liu et al., 2019). Angiopoietin-like 7 (ANGPTL7) belongs to a family of secreted angiopoietin-like proteins and plays vital roles in the modulation of hematopoietic stem cell maintenance, angiogenesis, and lipid metabolism (Zhang et al., 2006; Hato et al., 2008). Prior studies have revealed striking ANGPTL7 underexpression in various cancers such as colorectal cancer and breast cancer. Besides, upregulation of ANGPTL7 has been found in cancer cells after myeloid cell depletion, which affected liver metastasis by diminishing cell growth and vascular density, implying that ANGPTL7 could act as a mediator of metastatic progression and as a promising intervention target (Lim et al., 2015). As AGER and ANGPTL7 both show strong links to cancer, it is plausible that their interacted gene ATP13A4-AS1 also engages in the progression of lung cancer by exerting similar impacts on cellular activities. Furthermore, the Kaplan–Meier plot exhibited that lower levels of ATP13A4-AS1 correlated with poor patient outcome (Figure 6A), corresponding to its lower expression in LUAD than normal samples confirmed by our RT-qPCR (Figure 7A). Hence, our study revealed, for the first time, the roles of ATP13A4-AS1 in carcinogenesis, providing experimental clues for further investigations.

Dual oxidase 1 (DUOX1), an oxidant-generating enzyme within the airway epithelium, has been previously reported to take part in innate airway host defense and epithelial homeostasis, the activation of which stimulated cell migration dependent on Src family tyrosine kinases and epidermal growth factor receptor (EGFR) signaling pathways (Yoo et al., 2012; Gorissen et al., 2013; Sham et al., 2013; Hristova et al., 2016). DUOXA1, as a maturation factor of DUOX1, is transcriptionally and functionally linked to DUOX1 (Grasberger and Refetoff, 2006). Recent evidence has indicated that DUOX1 and DUOXA1 were frequently silenced due to promoter hypermethylation in various epithelial cancers including lung cancer (Luxen et al., 2008; Ling et al., 2014; Little et al., 2016). Consistent with pan-cancer analysis, DUOX1 and DUOXA1 were also downregulated in LUAD from our TCGA dataset, proving their potential tumor suppression roles.

HIG1 domain family member 1B (HIGD1B) is localized to the cell membrane whose expression is induced by hypoxia and glucose deprivation (Denko et al., 2000). Moreover, an increased expression of HIGD1B has been observed in cervical cancer and plurihormonal pituitary adenomas (Denko et al., 2000; Jiang et al., 2012). In contrast with previous research, HIGD1B showed lower expression levels in LUAD compared with normal samples in TCGA datasets, which was also observed in our LUAD patients and cell lines (Figure 7B), probably due to different cancer characteristics, implying its dual function in carcinogenesis. Moreover, its interaction with DUOX1 and DUOXA1 emphasized similar contributions to inhibit the malignant progress of cancer. The survival analysis also demonstrated that the low expression was associated with high risk (Figure 6B). Thus, our study expanded the roles of HIGD1B in cancer biology which need further validations.

In the medium orchid module, PIP5K1A, CRTC2, DAP3, and ISG20L2 deserved much attention for their central positions in the network (Figure 5B). As a lipid kinase, the basic function of PIP5K1A is to phosphorylate PI4P to synthesize important signaling phospholipid PI(4,5)P_2_, which serves as the substrate for phosphoinositide 3-kinase (PI3K) for conversion into PI(3,4,5)P_3_ to promote cell proliferation and survival (Adhikari and Counter, 2018). Intriguingly, PIP5K1A has been recently shown to interact directly with mutant KRAS and TP53, which were the most prevalent drivers in lung cancer, and facilitated downstream oncogenic signaling (Adhikari and Counter, 2018; Choi et al., 2019).

CRTC2, belonging to the CREB-regulated transcription coactivator (CRTC) family, can bind the leucine zipper DNA-binding region of CREB which results in the enhancement of CREB transcriptional activity (Koo et al., 2005; Dentin et al., 2008). Recent studies have identified novel mutations and a high expression of CRTC2 in non-small cell lung cancer patients, leading to reinforced migration and invasion abilities of cancer cells (Shi et al., 2018; Rodón et al., 2019). Current evidence has pointed out oncogenic roles of CRTC2 in lung cancer, consistent with its elevated expression levels in our study.

As a molecule involved in controlling apoptosis and anoikis, death-associated protein 3 (DAP3) has been highly implicated in the context of carcinogenesis. However, controversy exists with regard to its roles in human cancer (Wazir et al., 2015). Increased expression levels of DAP3 have been observed in invasive glioblastoma as well as glioma cells with induced migratory phenotype (Mariani et al., 2001), while an inverse association between DAP3 expression and clinical outcome has been found in breast cancer, corresponding to the pro-apoptotic function of DAP3 (Wazir et al., 2012). A recent study has identified differentially expressed DAP3 for classification between LUAD and normal samples (Hsu et al., 2015), indicating its crucial roles in carcinogenesis. Consistently, our Kaplan–Meier analysis showed better survival in the low transcription group approaching significance (Figure 6C), supporting tumor promotion influences of DAP3 on LUAD. At the same time, our experiments also witnessed the upregulation of DAP3 in LUAD patients and cell lines (Figure 7C). Therefore, our study illustrated that, for the first time, DAP3 occupied the core position in the regulatory network of LUAD, explaining its mechanism of action to some extent.

Interferon-stimulated 20-kDa exonuclease-like 2 (ISG20L2) is a novel vertebrate nucleolar exoribonuclease and involved in ribosome biogenesis (Couté et al., 2008; Simabuco et al., 2012). As one of target genes regulated by miR-139-3p, ISG20L2 has recently been related to hepatocellular carcinoma prognosis (Zhu et al., 2019), consistent with an immunogenomic landscape study which adds the roles of ISG20L2 in reflecting levels of infiltration in diverse immune cells (Xiao et al., 2021). Additionally, high expression of ISG20L2 has been found in several cancers and affected cancer occurrence in a variety of aspects (Xu et al., 2020; Xiao et al., 2021). Nevertheless, there is scant literature with regard to its role in LUAD. Here, we proposed for the first time the roles of ISG20L2 in promoting carcinogenesis and identified its interacted genes in the regulatory network which contributed to understanding further functional mechanisms. Accordingly, survival time was significantly higher in patients with low ISG20L2 expression, compared with the high ISG20L2 expression group (Figure 6D). Meanwhile, remarkable elevated levels of ISG20L2 were also verified by RT-qPCR in LUAD patients and cell lines (Figure 7D).

## Discussion

Although multiple studies have continuously explored gene interaction networks which were constructed from a series of correlated gene pairs, little is known about the differential correlations between normal and tumor status which contributed to elucidating deep mechanisms of gene regulation and identifying master genes. Benefiting from the availability of high-throughput data and different kinds of algorithms, detecting correlation changes became feasible. In this study, we developed an *in silico* framework to identify key genes with differential correlations in LUAD. Both candidate genes and their targets from gene regulatory networks could be further used for experimental investigations of biological functions in order to guide the diagnosis and treatment of patients. As transcriptomic data only represents a single layer of genome and is not sufficient to depict the whole-cell atlas, multidimensional molecular data including proteomic and metabolomic data as well as genome-wide association studies are required to be integrated for elaborating transition mechanisms from normal to tumor. Meanwhile, a statistical evaluation should be more strongly emphasized in future studies of differential correlations.

## Data Availability Statement

The datasets presented in this study can be found in online repositories. The names of the repository/repositories and accession number(s) can be found in the article/Supplementary Material.

## Ethics Statement

The studies involving human participants were reviewed and approved by The Third Affiliated Hospital of Soochow University. The patients/participants provided their written informed consent to participate in this study.

## Author Contributions

YoZ conceived the study. YiZ and JL conducted the database search and analysis. BX, TH, and XZ designed the algorithm and network construction. YL and HD conducted the experimental validations. YoZ and JJ wrote the manuscript. All authors contributed to biological analysis, interpretation of the results, read, and approved the final version of the manuscript.

## Conflict of Interest

The authors declare that the research was conducted in the absence of any commercial or financial relationships that could be construed as a potential conflict of interest.

## Footnotes

1 https://portal.gdc.cancer.gov/

2 https://string-db.org/

## Supplementary Material

Supplementary Figure 1 Module-trait associations. Each row corresponded to a module eigengene, column to a trait. Each cell contained the corresponding correlation and *P*-value. The table was color-coded by correlation according to the color legend.

Supplementary Figure 2 A scatterplot of Gene Significance (GS) for Disease vs. Module Membership (MM) in the yellow **(A)** and mediumorchid **(B)** modules. There was a highly significant correlation between GS and MM in the module.

Supplementary Table 1 The TCGA sample IDs and their clinical information.

Supplementary Table 2 Primer sequences of GAPDH, ATP13A4-AS1, HIGD1B, DAP3, and ISG20L2.

Supplementary Table 3 The members of each module.

Supplementary Table 4 Pair-wise differential correlations in the yellow module.

Supplementary Table 5 Pair-wise differential correlations in the mediumorchid module.

Supplementary Table 6 Oppositely correlated gene pairs with differentially expression simultaneously from the yellow and mediumorchid modules.
