# Supplementary figures and images for: Identification of Key Genes With Differential Correlations in Lung Adenocarcinoma
Source: Front Cell Dev Biol. 2021 May 5;9:675438. doi: 10.3389/fcell.2021.675438 (PMC8131847; doi:10.3389/fcell.2021.675438)

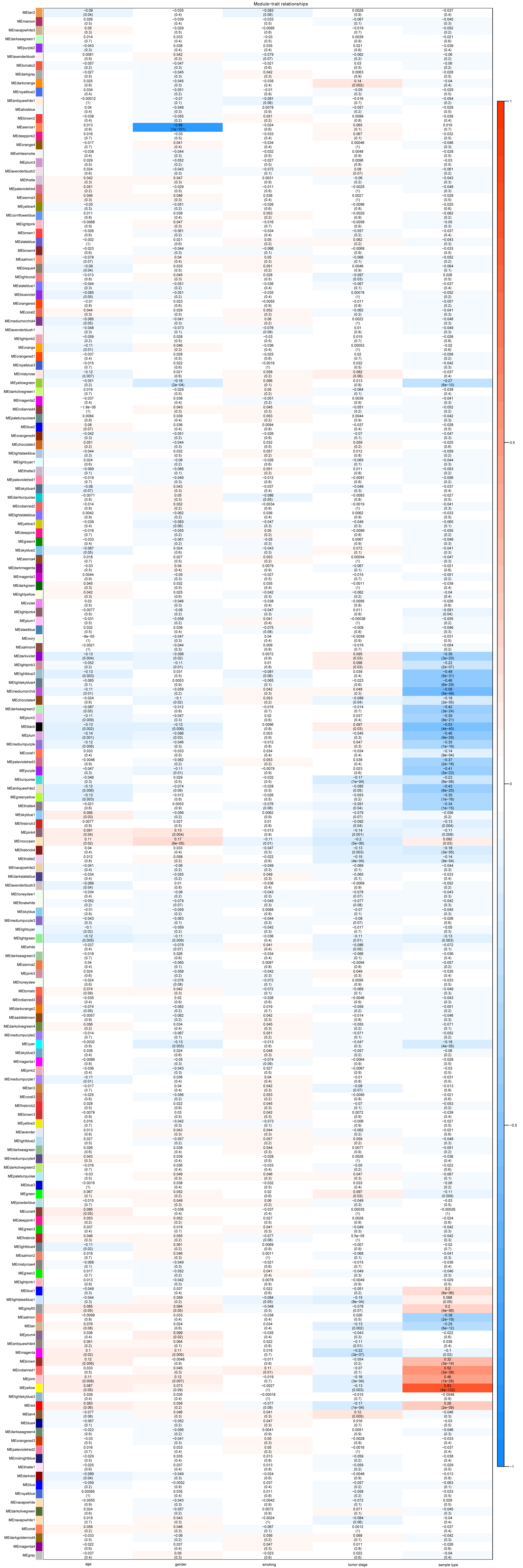

Supplement: Supplementary Figure 1 — Module-trait associations. Each row corresponded to a module eigengene, column to a trait. Each cell contained the corresponding correlation and P-value. The table was color-coded by correlation according to the color legend. [file Image_1.TIF]

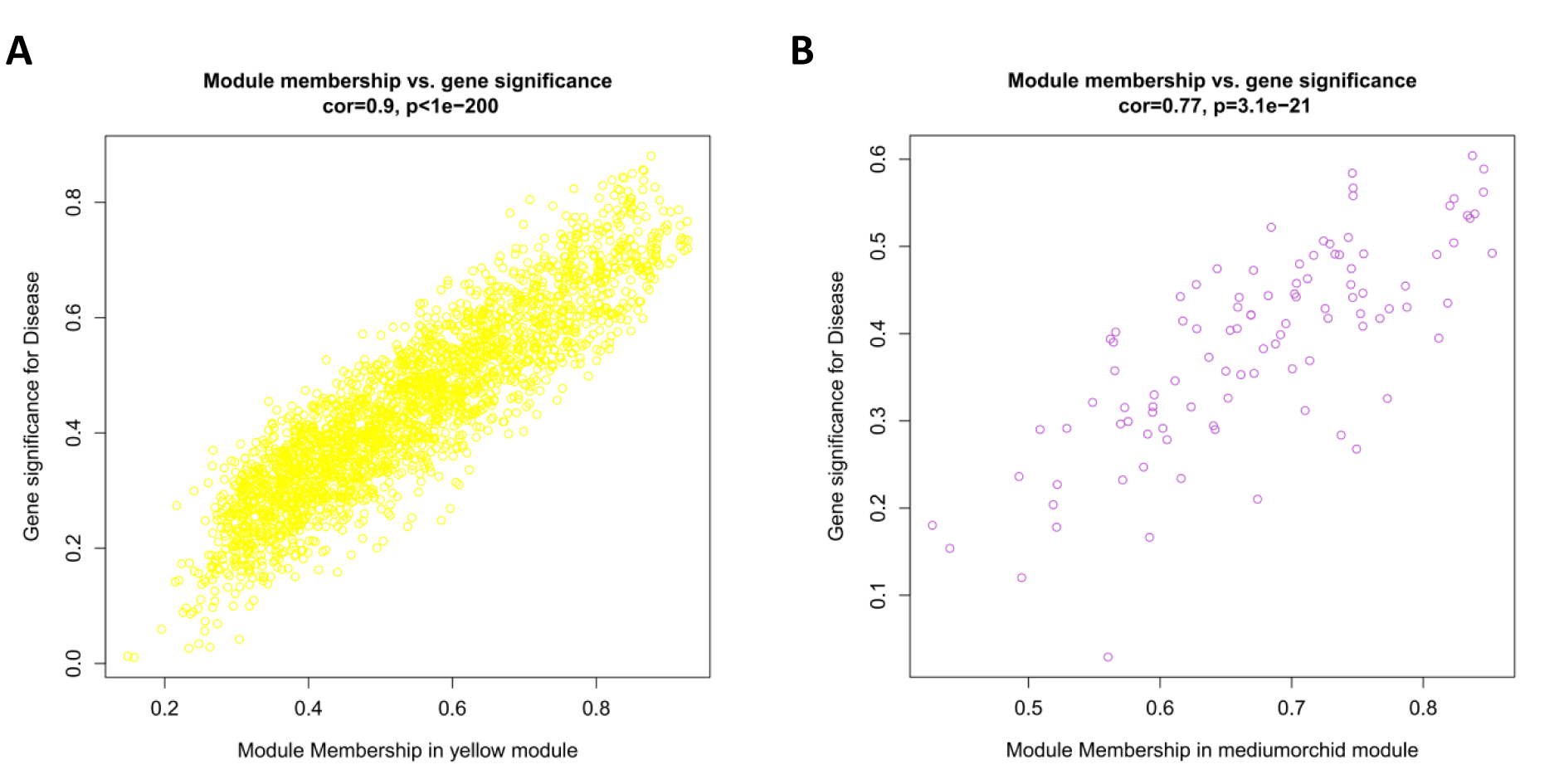

Supplement: Supplementary Figure 2 — A scatterplot of Gene Significance (GS) for Disease vs. Module Membership (MM) in the yellow (A) and mediumorchid (B) modules. There was a highly significant correlation between GS and MM in the module. [file Image_2.TIF]
